# Facilitators to doctoral completion among international students in a Global South University: An appreciative inquiry perspective

**DOI:** 10.1371/journal.pone.0340779

**Published:** 2026-01-30

**Authors:** Songcun Zhang, Hui Zhan

**Affiliations:** 1 School of Humanities and International Communication, Ningbo University of Technology, Ningbo, Zhejiang, China; 2 Patton College of Education, Ohio University, Athens, Ohio, United States of America; King Abdulaziz University Faculty of Medicine, SAUDI ARABIA

## Abstract

In the context of the global knowledge economy, the Global South has emerged as an increasingly important destination for doctoral education, yet research on this phenomenon remains scarce. The present study addresses this research gap by investigating the factors that contribute to the successful completion of doctoral programs among international students from the Global South enrolled at a public research university in Malaysia. Employing the Appreciative Inquiry approach, this study conducted semi-structured interviews with 11 international doctoral graduates. Findings reveal that intrinsic factors, such as passion for research, perseverance, and self-discipline, are crucial for success. External factors, including support from supervisors, institutions, families, and friends, also play a significant role. Furthermore, the Global South participants in this study called for improving conditions for future cohorts and fostering cross-cultural understanding within South-South doctoral education, where resource constraints and uneven institutional support often necessitate mutual aid and solidarity, a dimension largely overlooked in studies focused on Global Northern higher education systems with more robust infrastructure. The study contributes to understanding doctoral experiences in the Global South, challenges the dominance of a northern-centric perspective in academic research, and offers practical implications for institutions seeking to enhance support for doctoral students.

## Introduction

The “Global South” is a geopolitical, economic, and socio-cultural concept that refers to a diverse group of countries primarily located in Africa, Asia, and Latin America, which are mostly less economically developed and politically or culturally marginalized [1]. Based on Connell’s Southern theory (2013), colonial social orders exhibit inequalities in both politics and knowledge, with Northern hegemony persisting through textbooks, paradigms, and bibliographies in the Global South. Analyzing these global social orders and deconstructing North-South structural differences are critical to understanding societies shaped by colonialism and neocolonialism, and to constructing knowledge from the periphery [2]. It is urgent to challenge Western intellectual dominance in the Global South and develop new approaches to learning using globally expanded resources. Regions in the Global South have undergone educational restructurings to produce skilled graduates and innovative researchers for the global academic economy [3].

Some governments in the Global South, such as Malaysia, have sought to deconstruct Northern hegemony by introducing university ranking systems that focus on research productivity [2]. World rankings are widely regarded as benchmarks for university quality [4–5]. Malaysian universities have strategically improved their rankings to market programs, gain global recognition, and attract students, faculty, and funding [6]. These strategies include international collaborations with higher education institutions (HEIs) [6], boosting high-impact publications [7], and using English as the medium of instruction [8]. Educational credentialism drives many students, including those from China, to pursue higher education in Malaysian universities [9]. Xu et al. (2024) note that doctoral students in Malaysia leverage Southern niches to gain benefits, though their adaptive strategies may unintentionally reinforce Western dominance [10].

This study focuses specifically on doctoral students from the Global South at a public university in Malaysia for three reasons. First, this group is underrepresented in existing literature, which has historically centered on international students in the Global North (e.g., [11–12]). Yet, south-south student mobility—where students from Global South countries pursue education in other Global South nations—is growing rapidly, driven by shared cultural, linguistic, or economic ties [13]. Second, international students from the Global South face unique challenges in transnational doctoral education, including navigating hybrid academic norms, blending Western research paradigms with local knowledge systems, and balancing financial constraints with high academic expectations [10]. Third, Malaysia was chosen as the study site due to its distinct role as a hub for South-South doctoral education, setting it apart from other Global South countries. Malaysia’s primary source countries for international students include China, Indonesia, Bangladesh, Pakistan, and Nigeria [14]. Most research on doctoral programs has centered on the Northern Hemisphere, with well-developed training systems [15]. In contrast, studies on doctoral education in the Global South, such as Malaysia, remain sparse [13].

To address the aforementioned research gaps, this study investigates the factors that enable doctoral students from the Global South to complete their programs on time at a public research university in Malaysia, employing the Appreciative Inquiry framework. As a methodological approach aligned with the principles of positive psychology, Appreciative Inquiry centers on identifying strengths and exploring potential pathways to facilitate individual and organizational development and change [16], making it useful for identifying solutions [17–19].

This study serves as a valuable guide for institutions and students alike in creating a supportive ecosystem for doctoral education in the Global South. It also contributes to the literature on the experiences and perspectives of international doctoral students in universities in the Global South, extending beyond the overall northern-centric pattern of global knowledge production. The research question is: What factors contribute to doctoral students from the Global South overcoming challenges and completing their programs?

### Literature review challenges for doctoral student graduation

Pursuing a doctoral (Ph.D.) degree constitutes a complex and challenging endeavor. Specifically, social contextual factors, institutional dynamics, and individual-level characteristics operate as an interconnected complex system that influences the successful completion of doctoral programs [20].

A key area of challenge is doctoral students’ mental health and well-being [21–22]. In Evans’ research in the U.S., 90% of graduate students reported experiencing anxiety and depression, which was six times higher than the general population [23]. Meanwhile, around 25% of British doctoral students were experiencing poor well-being [24]. These figures identified poor mental health and well-being among postgraduate students as a critical challenge to doctoral student attrition [25–27]. The surge in online learning adds another layer of complexity, posing more mental health challenges to students [28–29]. Recent studies state that international doctoral students with hybrid supervision may feel isolated and disconnected from the academic community, leading to a lack of motivation and engagement [30–31]. This hybrid supervision can bring emotional and mental health challenges, as international students may face increased stress and anxiety due to independent research and online guidance. The absence of emotional support and academic guidance can exacerbate these challenges and lead to dropout.

Time management emerges as another pivotal challenge for doctoral students [32]. A qualitative study highlights that deficiencies in time management skills, reduced motivation, and insufficient supervisor-student interaction are the main challenges impeding the successful completion of doctoral programs by international students [33]. Another case study with nontraditional students, the entry-level occupational therapy doctoral students, also highlights the importance of time management [34]. This challenge is especially true for mature postgraduate students with family, social commitments, and other employment commitments, such as university lecturers or college administrators, in addition to their studies abroad. Balancing academic work with research obligations while studying outside their home countries poses significant challenges for international doctoral students seeking to maintain concurrent employment.

Furthermore, international doctoral students face significant cultural and language challenges [35–36]. Fanari and her colleagues identify language and cultural differences as key challenges international students face in the United States [37]. These challenges encompass various aspects, including communication styles, academic expectations, and social norms. For instance, navigating academic writing conventions and adapting to discourse practices in a different cultural context can pose considerable hurdles. Similarly, Elliot and Kobayashi (2018) highlight that international doctoral students encounter difficulties not only with language proficiency but also in cultural adaptation and academic writing [38].

Additionally, challenges in cultural adaptation and academic writing affect international students in advanced countries as well as students transitioning between countries in the Global South. Malaysia, as an international educational hub in the Global South, has a doctoral education that is mainly research-focused and has high-quality requirements. Most Malaysian doctoral programs require students to take four or six credit hours in methodology-related courses and one Bahasa Melayu course, then finish a high-quality dissertation in 60,000–100,000 English words to pass the final defense and at least two academic publications in worldwide acceptant journals [39]. Hence, academic writing is obviously important for doctoral students in Malaysia [40].

Other studies focus more on the doctoral supervisor-student relationship, and authors believe it is one of the critical challenges for completing doctoral programs [39,41]. The misalignment of expectations between doctoral supervisors and their students is identified as a significant challenge contributing to high attrition rates and delayed completion of doctoral programs among students at Malaysian universities. A mixed-method study reveals a significant perception difference between supervisors and international postgraduate students due to cultural differences, leading to misunderstandings, reduced engagement, and decreased affordance [42]. Most of the studies mentioned above are Western-centric perspectives, which originated from doctoral students’ experiences and realities of the Global North. There is a need to encourage and investigate the diversified experiences and perceptions of international doctoral students from the Global South.

### Factors contributing to doctoral student completion

While doctoral enrollment at Malaysian universities has experienced a significant surge, there remains a paucity of scholarly studies examining two key areas: first, how doctoral students from the Global South adapt to the local academic and social contexts; and second, the challenges associated with insufficient motivation and language-related barriers [10,33,43]. Therefore, further research is needed on the successful experiences of Global South students pursuing doctoral degrees in Malaysia.

The existing literature has examined the educational journeys of Global South students pursuing studies in the Global North, highlighting challenges such as cultural adaptation, academic integration, and systemic barriers [11,12,44]. A systematic review identifies three primary positive factors that support students’ psychological well-being, which contribute to successful doctoral study processes: self-care and lifestyle, support networks, and scholarly community support [45]. Specifically, exercise stands out as the most critical component of an effective healthy lifestyle, as it directly alleviates academic stress and enhances emotional resilience [46]. Beyond individual practices, caring relationships with family and friends form the foundation of a support network, providing doctoral students with emotional stability and practical assistance amid rigorous academic demands [46]. Besides, supervisors’ support plays a pivotal role in fostering a cohesive scholarly community, creating a safe space for students to seek guidance and feel intellectually supported [46–47]. Together, these factors highlight the multidimensional nature of support needed to sustain doctoral students’ well-being.

Several Malaysian-focused studies have explored factors influencing master’s and doctoral completion. A quantitative study of 320 international students at a Malaysian public research university identified six core contributors to graduation: supervision arrangements, research skills, research work, institutional factors, motivational factors, and demotivational factors [48]. Notably, the findings highlighted academic ability as the most critical predictor of on-time doctoral completion. Complementary research on international students in Malaysia further underscores the significance of academic capability, alongside contextual supports, for degree completion [49–51]. For instance, Singh’s (2021) study, based on 33 semi-structured interviews, found that group assignments, in-classroom practices, and university support helped international students develop resilience and progress toward graduation. Similarly, a quantitative analysis of 191 Malaysian international students [50] confirmed that research skills, self-management, institutional support, and supervisory practices were key motivators for on-time completion. Yet, these studies failed to distinguish doctoral students from master’s students, blurring insights specific to the doctoral journey.

The issue of international doctoral students’ graduation on time is under-researched. Few studies have analyzed the links between factors and academic success among this group; most existing research focuses broadly on international student populations [52]. This study addresses the aforementioned research gap by focusing on international doctoral students from the Global South and examining the factors that facilitate their timely completion of doctoral programs, with the Appreciative Inquiry framework serving as the overarching methodological guide.

## Methodology

### Appreciative inquiry approach

Max Cooperrider and his advisor, Suresh Srivastva, first laid out the basic framework of Appreciative Inquiry in their original articulation, *Appreciative Inquiry in Organizational Life* [53]*.* Rather than focusing on deficits, the Appreciative Inquiry approach was used to explore strengths and success factors, aligning with its core principle of driving positive change through the analysis of what “works well” [18]. It contains four steps: Discovery, Dream, Design, and Destiny [54–55]. Discovery involves identifying and gathering stories, experiences, and examples of what has worked well on one issue in the past. The focus is on framing the problem positively. In the Dream step, individuals within the organization are encouraged to envision the future they want to create. The focus is on generating a shared vision that is compelling and inspiring. Design involves developing specific strategies and plans to achieve the shared vision. The final step, Destiny, involves continuous learning and improvement. The focus is on maintaining momentum and making ongoing improvements to achieve the shared vision.

There are several reasons for selecting the Appreciative Inquiry approach in this study. First, it is expected to promote positive change. Different from traditional problem-solving methods, the Appreciative Inquiry approach focuses on discovering and enhancing the strengths and successful experiences of organizations or individuals as the basis for promoting change [56]. This approach has the potential to foster a more positive work culture and enhance individual levels of engagement. Second, it is possible to uncover collective intelligence. Through active collaboration, an Appreciative Inquiry approach can stimulate collective intelligence and build team capabilities.

### Participants

Participants in this study were international doctoral graduates from the Global South who had completed their degrees at a Malaysian public research university. This study used criterion sampling [57] to recruit participants, with inclusion criteria: (1) status as an international student from a Global South country at a Malaysian public university; (2) on-time completion of a doctoral program (within five years, per Malaysian university regulations); and (3) graduation between 2021 and 2024. Purposive sampling refers to researchers intentionally selecting participants based on their certain characteristics, knowledge, experiences, or other criteria [57–58].

Invitations were sent via WhatsApp to 53 international doctoral students from the 2021–2024 Bahasa Melayu class groups, as these groups included recent graduates with shared experiences of the university’s doctoral program. Of 18 responses, 11 participants met the criteria and agreed to participate in this study. The 11 participants included six females and five males, with an average age of 35.5 years and an average time to completion of 3.6 years. Their countries of origin were China (6), Pakistan (3), Sri Lanka (1), and Indonesia (1), and most majored in social sciences, such as Education, Business, and Economics. Pseudonyms were used to protect confidentiality (See Table 1).

**Table 1:** Interviewee information.

Name (gender)	Age	Duration	Country	Faculty	Graduate
Daisy (female)	33	5	China	Education	2024
May (female)	34	5	China	Education	2023
Max (male)	37	4	China	Engineering	2022
Wilson (male)	34	4	China	Business and Economics	2023
Ng (male)	28	3	China	Computer Science	2023
Han (male)	40	3	China	Language and Linguistics	2024
Fayra (female)	37	3	Pakistan	Business and Economics	2023
Anna (female)	39	3	Pakistan	Art and Social Science	2021
Mirza (male)	38	4	Pakistan	Education	2022
Ina (female)	35	3	Sri Lanka	Education	2023
Lee (female)	39	3	Indonesia	Management	2021

### Instrument

For this study, Appreciative Inquiry’s four-stage framework (Discovery, Dream, Design, Destiny) guided the research design, data collection, and analysis to ensure a structured exploration of participants’ successful doctoral journeys. For the discovery stage, we explored the past successes and strategies used by participants to overcome challenges. For the dream stage, we tried to identify participants’ aspirations and visions for effective doctoral education. For the design stage, we tried to develop actionable strategies to support future doctoral students. For the destiny stage, it is to envision long-term outcomes of sustained success in doctoral education. This framework informed the development of semi-structured interview questions (See Appendix A), which were designed to elicit reflections on strengths and support systems—ensuring alignment with Appreciative Inquiry’s focus on positive inquiry (See Table 2).

**Table 2:** Mapping interview questions to appreciative inquiry stages.

Stages	Interview questions (Appendix A)	Rationale for Alignment
Discovery	Q2, Q3, and Q5 to identify strengths in the Global South doctoral journeys	Probe past successes/ strategies
Dream	Q1, Q4, and Q5 (aspirations for equitable education)	Elicits aspirations for self/others
Design	Q6 (contextual supports for success)	Explores strategies to realize dreams
Destiny	Q7 (sustained growth in resource-constrained settings)	Links strengths to long-term growth

### Data collection

This study received ethical approval from the Research Ethics Committee of the School of Foreign Languages at Ningbo University of Technology, Ningbo, Zhejiang Province, China (Reference Number: NBUTSFL202504010002). Before data collection, all participants were thoroughly informed about the study’s purpose, procedures, and confidentiality measures. Oral consent was confirmed at the start of each interview to document the participant’s agreement to the audio recording.

Data collection was conducted over one month in May 2025. Interviews were conducted by the first author (female, a faculty member at a local university in the United States), a researcher with experience in qualitative methods and doctoral education research. All interviews were held online via Microsoft Teams, conducted in English, and audio-recorded for later transcription. Each interview lasted approximately 40 minutes at the workplace, following the semi-structured guide in Appendix A to ensure consistency while allowing flexibility to probe emerging themes. Audio recordings were transcribed verbatim by a professional transcriber fluent in academic English, with the first author cross-checking 20% of transcriptions against recordings to ensure accuracy. Transcripts were anonymized before analysis, removing the names of participants, supervisors, and institutions.

### Data analysis

Deductive thematic analysis, a theory-driven data interpretation method that extracts themes from data that align with an existing theoretical framework or a pre-set research question [59], was used in this study. Deductive coding was guided by Appreciative Inquiry’s four stages, with pre-defined codes. For example, the Discovery stage means finding strengths used to overcome challenges in the doctoral journey. After initial coding, inductive thematic analysis techniques were integrated to facilitate open coding both within and beyond the pre-established categories, enabling the identification of prominent themes not fully foreseen by the theoretical framework. This approach ensured the analysis maintained theoretical grounding while remaining responsive to the unique content of the themes and subthemes.

### Findings

The findings, organized by Appreciative Inquiry’s four stages (Discovery, Dream, Design, and Destiny), highlighted the strengths, strategies, and support systems that enabled doctoral students from the Global South to complete their programs successfully. A summary of main themes and sub-themes was provided in Table 3.

**Table 3:** Summary of findings.

Stages	Main Themes	Sub-themes
Discovery	Foundational strengths in navigating doctoral demands	Lifelong learning and knowledge acquisition Intrinsic motivation Emotional resilience Proactive communication with supervisors Adaptability to emergencies
Dream	Aspirations fueled by past success	Pride in academic achievement Desire for intercultural collabor ation Advocacy for resource equity
Design	Strategic supports for sustained progress	Institutional infrastructure and guidance Peer and family networks
Destiny	Long-term growth through intrinsic motivation	Autonomy and self-efficacy Personal and professional development

### Discovery stage

The Discovery stage identified core strengths that participants leveraged to navigate the rigors of doctoral education. These strengths were actively applied to address the demands of doctoral work, from bridging knowledge gaps to managing unexpected challenges.

One crucial strength was a commitment to lifelong learning and continuous knowledge acquisition. Participants emphasized that doctoral study required ongoing effort to fill gaps between master’s education and doctoral expectations. As one participant, Han, noted, “Writing a doctoral thesis is a long journey that you must persist in your research and fill your knowledge gap between the master’s and doctoral stages. As knowledge is unlimited, you must devote yourself to learning and keep learning.” This dedication to growth was critical for mastering complex methodologies and academic writing conventions. People with doctoral degrees are usually considered knowledgeable and respected. It is not easy for them to obtain a large amount of knowledge. It usually takes three to six years to obtain their doctoral certificate. They need to persist in learning throughout the whole process. Han’s sharing demonstrated the importance of continuous knowledge acquisition in the doctoral journey, where success was achieved through dedication and a commitment to expanding the individual’s knowledge base.

Intrinsic motivation, such as passion for research and self-discipline, was identified as the “driving force” behind sustained progress. Participants emphasized that external supports mattered, but internal drive determined their ability to persist through setbacks. As Fayra stated, “I have an interesting topic, and I could enjoy the research process based on my interesting topic.” May also reported, “I have great passion for my research topic, and I consider that what I am doing is the most important. Without passion and interest, it is almost impossible to persist in your research for a long time.” Therefore, it is significant for doctoral students to choose research topics that interest them. Furthermore, all the interviewees confirmed that self-discipline and hard work were crucial in completing their doctoral education. Max stated, “Discipline yourself. Discipline leads to good time management, then good time management leads to success.” Fayra stated, “Doctoral students need to balance their work and life with their families.” Sometimes, your progress may be slow, but you should be consistent and hard-working (Mirza and Han). Intrinsic motivation is significant to doctoral students’ research development.

Emotional resilience was also central to success. Participants faced moments of stress, frustration, or self-doubt, but they employed intentional coping strategies to maintain momentum, such as exercising, peer discussions, or reflection to manage pressure. As Ina reported, “Emotional distress did occur. Sometimes, I felt upset during the long doctoral journey. I must do exercise to adjust myself.” Mirza also stated, “Sometimes I feel stuck, and I do not know what to write anymore. Such confusion is a challenge. Moreover, I feel frustrated when I have to revise the methodology chapter many times.” However, the focus here was on how students like Ina and Mirza developed coping plans, such as exercise and self-reflection, which helped them manage their emotions and made them stronger and more resilient in the transforming process of challenges to personal growth.

Proactive communication with supervisors emerged as another vital strength. Participants described strategies such as scheduling regular meetings, clarifying expectations, and seeking feedback early to foster productive relationships. Based on interview data, several interviewees highlighted that consistent interaction, whether weekly check-ins or draft reviews, helped align their work with academic standards and reduced misunderstandings. For example, Ina stated that her supervisors were helpful and supportive in teaching them how to publish journal papers, and they met every week to discuss her research topic. The positive interaction between students and supervisors played a crucial role in the successful completion of the doctoral journey. As Ina emphasized, doctoral students should be grateful to their supervisors, interact with them frequently, and maintain a positive relationship with them. It might be helpful for your research progress.

Adaptability to unforeseen emergencies further demonstrated participants’ strengths. Whether balancing family health crises, financial constraints, or logistical disruptions, participants prioritized flexibility. For example, Max recalled, “My father was seriously ill when I was writing my doctoral dissertation. But I adjusted my schedule—working early mornings and late nights—to meet deadlines. It was not easy, but my determination to finish kept me going.” It is highlighted that doctoral students have strength and adaptability in dealing with emergencies. Furthermore, they demonstrated remarkable resilience and determination to succeed when overwhelmed by the stress of academic writing and daily life.

In sum, participants did not merely cope with challenges; they actively deployed strengths—persistence, proactive communication, resilience, and adaptability—to transform obstacles into opportunities for growth. These strategies were foundational to their ability to progress through the doctoral journey.

### Dream stage

The Dream stage centered on participants’ aspirations, which were rooted in their experiences of overcoming challenges and achieving milestones. These aspirations reflected both personal pride in their accomplishments and a vision for improving doctoral education for others.

Pride in academic achievement was a universal sentiment. All participants described feelings of fulfillment upon completing their degrees, which reinforced their motivation to pursue further academic goals. This study started by discussing the interviewees’ feelings after passing their final defenses and being informed of their successful graduation. This focus aligns with the core principle of the Appreciative Inquiry approach: Participants gain inspiration by reflecting on their own real, positive experiences [54]. All the interviewees confirmed that they were happy and proud of themselves at that moment. As Daisy stated, “Hearing congratulations on my dissertation defense was validation of years of hard work. It made me eager to contribute more to my field.” This sense of accomplishment fueled dreams of publishing research, teaching, or leading projects that addressed global challenges.

A desire for enhanced intercultural collaboration also emerged. Participants recognized that their success depended on navigating diverse academic and cultural contexts, and they hoped future students would benefit from stronger cross-cultural support. Based on interview data, international doctoral students from different countries experienced challenges related to cultural differences and language barriers, including culture shock. As Daisy stated, “In mainland China, we were educated to respect our teachers. Doctoral supervision is more hierarchical, and students rarely question supervisors’ feedback. But in Malaysia, my supervisor encouraged me to debate methodology choices. I felt uncomfortable at first, then discussed this with a senior Chinese peer, who shared strategies to balance respect for hierarchy with academic criticality. This helped me improve my dissertation.” In addition, May reported, “The language barrier is a severe problem that needs to be solved, which is one factor leading to misunderstandings between international doctoral students and their supervisors. For instance, my supervisor initially misinterpreted my quietness in meetings as disengagement—when in reality, I was translating complex ideas into English and feared making mistakes. We resolved this by agreeing to written check-ins between meetings, which gave me time to articulate my thoughts clearly.” For Chinese native speakers, it is not easy for them to completely understand English academic expressions, especially at the beginning of their doctoral journey. They adapted to the English academic background through hard work. Those international students’ dreams included enhancing intercultural understanding for various uncertain situations and achieving meaningful contributions to their academic fields in the end.

Advocacy for resource equity was another key aspiration. Participants highlighted the need for increased funding for conferences, workshops, and research materials. As Mirza reported, “Many of us paid for workshops out of pocket, but not everyone can afford that. I hope future students have access to grants that let them focus on learning, not finances.” These dreams were not just personal; they reflected a commitment to creating a more supportive environment for the next generation of doctoral students.

The Dream stage of the Appreciative Inquiry framework centers on envisioning both the successful completion of one’s doctoral program and the positive outcomes associated with overcoming inherent challenges—specifically, personal growth and professional advancement. For international doctoral students, this envisioning process entails imagining a future state in which they can engage in effective communication and collaborative work with their supervisors, an outcome that would foster more robust and meaningful academic experiences.

### Design stage

The Design phase is to consider how “social architecture norms, values, structures, strategies, systems, patterns of relationship, ways of doing things … can bring … dreams to life” [56, p.283]. In this study, the Design stage focused on the systems and strategies participants identified as critical for sustaining success. These included institutional resources, social networks, and intrinsic motivation. All of which worked in tandem to support doctoral students’ progress and success.

Institutional infrastructure and guidance were foundational. Participants valued resources such as doctoral rooms, access to journals, and methodological workshops, which facilitated collaboration and skill-building. As Mirza stated, “Infrastructural support for doctoral students is important. For example, the doctoral room provided by some faculties is convenient and supportive for doctoral students.” Besides, supervisor support was framed as a key institutional strength. Max reported, “I participated in some projects with my supervisor. Collaboration with my supervisor made me see myself clearly as a researcher. It is good for my professional development.” Ina also stated, “My supervisor encouraged me a lot and contributed a lot to my whole doctoral journey.” It could be concluded that the close connection between doctoral students and their supervisors would be good for their research development.

Peer and family networks provided additional layers of support. Participants described relying on peers for technical advice, emotional encouragement, and accountability, whether through study groups or informal check-ins. Interviewees expressed their gratitude for support from families and friends (Daisy, May, Max, Mirza, Han, Wilson). As Mirza reported, “You’d better have a research team, not just for the academic support. Sometimes, you also need emotional support.” Family support, both financial and emotional, was especially critical for younger participants or those balancing work and study. As Ng noted, “My parents supported me financially when I could not work, and my peers kept me motivated during tough times. I could not have finished my doctoral dissertation without that network.” Thus, peer and family networks are essential factors to help them achieve their goals.

In conclusion, the factors leading to the success of international doctoral students, such as intrinsic motivation (passion for research, perseverance) and external factors (support from supervisors, universities, families, and friends), are used to design strategies and interventions that can help students’ success and achieve their ultimate goals.

### Destiny stage

The Destiny stage highlighted how participants’ strengths translated into lasting personal and professional growth, with intrinsic motivation emerging as the cornerstone of their long-term success. It’s about taking action on the designed plans and making the envisioned future a reality. For international doctoral students, this could involve implementing the strategies and support systems identified in the Design phase to ensure they could successfully navigate their doctoral journey and achieve their desired outcomes.

Participants emphasized that self-efficacy and autonomy, which were developed through navigating doctoral challenges, were critical for post-graduation success. They described a shift from relying on external validation, such as supervisor feedback, to trusting their own judgment. As Max stated, “Do not care about external factors too much. It is your intrinsic interest and self-motivation that help you go through the challenges and push you to complete your doctoral thesis. By the end, I learned to trust my research instincts, which has made me a more confident scholar.”

Beyond academic skills, participants noted profound personal growth. Doctoral study fostered resilience, critical thinking, and respect for diverse perspectives—qualities they attributed to overcoming challenges with intentionality. As Han reflected, “The process taught me to listen to others’ ideas, even when I disagreed.” Moreover, “When you give your opinion, you must use evidence to support your opinion. Those skills make me a better collaborator and person, not just a better researcher.” Not everyone will agree, and you need to be open-minded to the world around you.

Ultimately, participants framed their success as a product of intrinsic motivation rather than external rewards. Success in doctoral education is not just for academic improvement and career advancement but also for self-reflection, self-awareness, and making you a better person (Max, Han).

## Discussion

This study employed the Appreciative Inquiry framework to explore factors facilitating timely doctoral program completion among Global South students at a Malaysian public research university. The findings, structured around Appreciative Inquiry’s four stages (Discovery, Dream, Design, Destiny), reveal a multidimensional model of success rooted in the unique contextual realities of South-South student mobility.

### Intrinsic Motivation: The foundation of sustained success

A central finding was that doctoral students’ intrinsic motivation, encompassing passion for academic research, self-discipline, perseverance, and a commitment to lifelong learning, served as the cornerstone of participants’ success. To handle the challenges at a doctoral level, international students would require solid internal motivation. This aligns with prior research highlighting motivation as a key predictor of doctoral completion [47,48], but extends it by emphasizing its heightened importance in the Global South context.

These strengths were not merely passive coping mechanisms but active strategies to address context-specific challenges—such as bridging master’s-doctoral knowledge gaps, mitigating cultural misunderstandings in supervision, and balancing academic pressures with personal crises, such as family health issues or financial constraints. Notably, participants’ emphasis on “proactive communication” reflects a nuanced adaptation to hybrid academic norms in Malaysian universities, where Western research paradigms intersect with local supervisory practices. This extends beyond Northern-centric studies that often frame international student success as passive adaptation to Western institutional norms [11–12], highlighting the agency of Global South students in co-constructing supportive academic relationships.

Why did intrinsic motivation play such a central role? In the Global South, doctoral students often face fewer institutional resources, such as limited funding and smaller research teams, compared to their Global North counterparts [10]. This context places a greater burden on individual drive to compensate for structural gaps. Participants relied on their own initiative to seek out skills training, network with peers, and stay motivated—qualities that became even more critical in environments where external support was inconsistent.

### External Support Systems: Amplifying strengths

While intrinsic motivation was foundational, external support from supervisors, institutions, peers, and families amplified participants’ strengths and accelerated progress. These supports functioned as “enablers,” reinforcing motivation and reducing barriers to success.

At the Design stage, success was sustained by a synergistic interplay of institutional support, such as doctoral rooms, methodological workshops, supervisor mentorship, peer/family networks, and intrinsic motivation, such as research passion and self-discipline. Unlike Northern-centric literature that prioritizes individual factors (e.g., 32) or institutional resources in isolation (e.g., 41), this study highlights the interdependence of external support systems and internal drive in the Global South context. For example, peer networks provided emotional resilience, addressing language barriers and social isolation—challenges exacerbated by Malaysia’s English-medium instruction and cultural diversity among Global South students. Institutional support, particularly supervisor collaboration on research projects, served as a bridge between Western research standards and local knowledge contexts, a critical adaptation not captured in studies of Northern universities.

The findings of this part agree with some previous studies [40,60] that developing supportive relationships with supervisors, tutors, peers, and families is very helpful for students’ academic development. Peers provided camaraderie and troubleshooting, such as sharing software tips, while families often covered living expenses or cared for dependents—critical in contexts where doctoral stipends may be limited. Similarly, Benjamin et al. (2017) identified social support as a key predictor of doctoral well-being, but this study highlights its unique role in the Global South, where extended family networks often serve as primary safety nets. For example, many young Chinese doctoral students start their doctoral sojourn in their twenties immediately after finishing their Master’s degree. It is their family that supports them financially. Naturally, these doctoral students are grateful for their parents’ support.

### Unique Insights from the Global South

The findings of this study highlight the agency of Global South doctoral students—individuals who thrive by combining intrinsic motivation with creative use of limited resources. By focusing on strengths rather than deficits, this study also offers a more hopeful vision of doctoral education in the Global South. Participants did not just survive their programs; they grew personally and professionally, developing skills, such as adaptability and cross-cultural competence, that are invaluable in an increasingly global academic landscape. Their success stories underscore the potential of Global South universities to cultivate successful doctoral graduates, provided that intrinsic motivation is nurtured and external supports are made more accessible.

Furthermore, participants in this study consistently advocated for increased access to funding for conferences, workshops, and research materials, framing these resources as critical for overcoming structural disadvantages. For example, many participants paid for methodological workshops out of pocket, noting that financial barriers prevented them from accessing the same professional development opportunities as their Northern counterparts. Their aspirations were not just personal but collective. They sought to create a more equitable environment for future Global South students, reflecting a commitment to decolonizing doctoral education by addressing systemic inequalities. This advocacy orientation stems from the recognition that success in the Global South is often constrained by structural factors (e.g., limited institutional funding, unequal access to global academic networks) rather than individual deficits—an insight that Northern-centric studies, rooted in contexts of greater resource abundance, fail to capture. International doctoral students in South-South mobility who face resource constraints and uneven institutional support often necessitate mutual aid and solidarity.

## Conclusion

This study set out to identify the reasons why international doctoral students from the Global South completed their degrees on time at a Malaysian public research university. Intrinsically, students’ success stemmed from their passion for research, self-discipline, and a commitment to lifelong learning. These internal drivers enabled students to navigate knowledge gaps, manage setbacks, and maintain progress even when faced with competing demands. Externally, support systems amplified these intrinsic strengths, including regular guidance from a supervisor, institutional resources, peer networks, and family support. Together, these factors created a cycle of success: intrinsic motivation led students to actively seek out and leverage external supports, while those supports reinforced their drive to persist. This interplay was particularly adaptive in the context of a Malaysian public university, where students often balanced global academic standards with resource constraints, relying on both personal agency and community to meet milestones. In sum, on-time completion for these doctoral students was not accidental but the result of intentional effort—fueled by internal passion and discipline, and sustained by purposeful engagement with available external supports.

### Implications

The implications of this study include enhancing academic support, improving communication channels, addressing personal and emotional support, language support, and cultural integration. Firstly, institutions need to provide targeted academic support to bridge the knowledge gap between the master’s and doctoral stages. This set of targeted supports may include workshops, seminars, and structured mentorship programs, each designed to focus on building core competencies in research methodologies, academic writing, and literature review skills. Secondly, it is crucial to establish clear and effective communication channels between doctoral students and their supervisors. Regular meetings, accessible communication platforms, and training for supervisors on cross-cultural communication can help mitigate misunderstandings and foster a supportive relationship. Furthermore, it is imperative to establish a multifaceted support community that encompasses not only academic stakeholders, such as supervisors and peer doctoral students, but also non-academic support networks, including family members and friends. This can be facilitated through networking events, family engagement programs, and recognition of the role of social support in the doctoral journey. Thirdly, it is necessary to recognize the emotional challenges faced by doctoral students and provide resources such as counseling services, stress management workshops, and peer support groups to help students cope with personal negativity and maintain mental health. Finally, given the cultural differences and language barriers, institutions should offer cultural orientation programs and language support services to help international students adapt to the academic environment. This could include English language courses tailored to academic contexts and cultural sensitivity training for staff and faculty.

For international doctoral students, the study recommends maintaining a strong work ethic while cultivating a balanced degree of self-reliance, avoiding over-reliance on supervisors or institutional support systems. Consistent with this guidance, the findings of this study indicate that success in doctoral education is primarily rooted in four interrelated personal attributes of doctoral students: a genuine passion for research, intrinsic motivation, sustained diligence, and resilience.

### Limitations

There are several limitations in this study. First, the sample, though achieving data saturation, was demographically constrained. Most participants were from China and Pakistan, with a smaller representation from Sri Lanka and Indonesia. This narrow focus may constrain the generalizability of the study’s findings to international doctoral students from other regions of the Global South. Second, the study focused exclusively on students who completed their degrees on time. By excluding those who faced delays or attrition, it may underrepresent the full spectrum of experiences that influence doctoral progression. Comparing on-time completers with those who took longer could reveal additional nuances in how strengths and supports interact with barriers. Third, the Appreciative Inquiry framework, while effective for centering strengths, may have implicitly oriented participants toward discussing positive experiences. Though interviews were designed to elicit honest reflection, the focus on “what worked well” could have minimized discussion of more ambivalent or challenging moments, even as participants framed these as opportunities for growth. Finally, the study relied solely on interview data, with no triangulation from institutional documents, supervisor perspectives, or other quantitative data. Triangulation would have strengthened the validity of themes by cross-verifying participants’ accounts with contextual data. These limitations underscore the necessity for future research to address three key areas: first, expanding the demographic scope of the sample; second, incorporating doctoral students who completed their programs with delays; third, triangulating self-reported data with longitudinal or observational methodologies. Such methodological and sampling refinements are critical to advancing a more comprehensive and nuanced understanding of doctoral program success among students in the Global South.
